# Evaluating the effects of anticoagulants on *Rhodnius prolixus* artificial blood feeding

**DOI:** 10.1371/journal.pone.0206979

**Published:** 2018-11-29

**Authors:** Lívia Silva-Cardoso, Felipe A. Dias, Patricia Fampa, Miria G. Pereira, Georgia C. Atella

**Affiliations:** 1 Instituto de Bioquímica Médica Leopoldo de Meis, Universidade Federal do Rio de Janeiro, Rio de Janeiro, Rio de Janeiro, Brazil; 2 Departamento de Ciências Farmacêuticas, Instituto de Ciências Biológicas e da Saúde, Universidade Federal Rural do Rio de Janeiro, Seropédica, Rio de Janeiro, Rio de Janeiro, Brazil; 3 Instituto de Biofísica Carlos Chagas Filho, Universidade Federal do Rio de Janeiro, Rio de Janeiro, Rio de Janeiro, Brazil; Instituto Oswaldo Cruz, BRAZIL

## Abstract

Blood-sucking insects are responsible for the transmission of several important disease-causing organisms such as viruses, bacteria, and protozoans. The hematophagous hemipteran *Rhodnius prolixus* is one of the most important vectors of *Trypanosoma cruzi*, the etiological agent of Chagas disease. Due to the medical importance of this insect, it has been used as a study model in physiology and biochemistry since the 1930s. Artificial feeding has been recognized as a feasible and a more ethical alternative method of feeding these hematophagous insects. To prevent clotting after blood collection defibrination or treatment with anticoagulants are necessary. Although anticoagulants have been routinely used for stabilizing the collected blood, there is a gap in demonstration of the effects of using anticoagulants on the feeding and development of the hematophagous insect *Rhodnius prolixus*. In this study, we compared the survival rate, molting efficiency, fertility, and infection development between insects that were fed on blood containing three different anticoagulants (citrate, EDTA, and heparin). We observed that fifth instar nymphs that were fed on blood containing EDTA and citrate could not perform digestion properly, which resulted in molting inefficiency. Adult insects that were fed on EDTA-containing blood laid lower number of eggs, and also had a diminished egg hatch percentage. When we delivered *T*. *cruzi* parasites in blood containing citrate or EDTA to the insects, a lower number of parasites and metacyclic trypomastigotes was observed in the intestine compared to the group fed on heparin-containing blood. Since heparin could potentially inhibit DNA polymerase activity in DNA samples extracted from the intestine, we analyzed different heparin concentrations to determine which one is the best for use as an anticoagulant. Concentrations ranging between 2.5 and 5 U/mL were able to inhibit coagulation without severely impairing DNA polymerase activity, thus indicating that this should be considered as the range of use for feeding experiments. Our results suggest that among the three anticoagulants tested, heparin can be recommended as the anticoagulant of choice for *R*. *prolixus* feeding experiments.

## 1. Introduction

More than one million species of insects have been described, exhibiting the most diverse eating habits and lifestyles [1]. Among that diversity, approximately 14,000 species have developed the ability to feed on vertebrate blood [2]. Adaptation of hematophagous arthropods to a blood-feeding environment thus entails specific morphological, physiological and behavioral adaptations that allowed these animals to attain and digest the nutrients present in that diet [3,4].

Blood-sucking insects are responsible for the transmission of several important disease-causing organisms such as viruses, bacteria, and protozoans [3]. In an attempt to reduce the health impact of insect vectors, researchers of different knowledge areas began investigating the biology of such insects, which led to the emergence of a field of study known as medical entomology. The biological knowledge about vector species has resulted in the development and application of different strategies to reduce the likelihood of transmission of disease-causing organisms. These strategies have focused on reducing population sizes and limiting human contact, as well as in the elimination of vector-breeding sites and developing chemical insecticides, insect repellents, and physical barriers [5,6]. More recently, genetic information and genome-editing technologies have been applied in vector control initiatives to reduce the competence or population size of vectors [7–9].

The hematophagous hemipteran *Rhodnius prolixus* is one of the most important vectors of *Trypanosoma cruzi*, the etiological agent of Chagas disease. Approximately 6–8 million people are infected with *T*. *cruzi* in the world, predominantly in the endemic area of Latin America, where it is primarily vector-borne and transmitted to humans through contact with feces or urine of triatomine bugs. Chagas disease has become a worldwide concern because of the increase in global population movements of infected subjects to non-endemic areas such as North America and Europe. In the absence of natural vectors, the parasite can be transmitted through blood transfusion, organ donation or by vertical transmission from mother to child [10].

Considering the medical importance of *R*. *prolixus*, this insect has been used as a model for studying insect physiology and biochemistry. These studies have helped understanding the mechanism of parasite transmission and in developing vector control strategies. The first laboratory colony was established in 1930, and these original strains has been maintained until today [11,12]. Studies on *R*. *prolixus* have investigated the gorging of blood meal [13–16] the effects of larval nutrition on the egg production of an adult [17], the growth and reproductive performances using different blood sources [18], the orchestration of endocrine events related to feeding [19], the effect of *T*. *cruzi* on *R*. *prolixus* life’s history [20], and its genome sequencing [21].

Triatomines develop better when fed on live animals, although this is not always feasible and ethical. Besides, artificial feeding facilitates some kinds of entomological studies, basic physiology, vector-pathogen interaction, and drug discovery and efficacy. Given its conveniences an in vitro feeding system is an alternative method used to provide the insects with freshly drawn blood of animals. Several studies have demonstrated the successful development and use of different devices to artificially feed blood-sucking invertebrate vectors of human pathogens [22–25]. Most of these approaches share some common features; in general, blood is placed between a heating element (used to mimic vertebrate blood temperature) and a thin membrane, into which the insects penetrate using their proboscis to access and imbibe the blood. [18,26–30]

After collection it is necessary to defibrinated or treated blood with anticoagulants to prevent clotting. Despite the routine use of anticoagulants for blood collection all around the world, there is a gap on the effects of using anticoagulants on the feeding and development of the hematophagous insect *Rhodnius prolixus*. In the present study, we analyzed the effect of three different anticoagulants (citrate, EDTA, and heparin) used during artificial blood feeding in physiologic and reproductive parameters of *R*. *prolixus*.

## 2. Materials and methods

### 2.1. Insects and ethical statement

All animal care and experimental protocols were conducted following the guidelines of the institutional animal care and use committee (Committee for Evaluation of Animal Use for Research from the Federal University of Rio de Janeiro, CAUAP-UFRJ) and the NIH Guide for the Care and Use of Laboratory Animals (ISBN 0-309-05377-3). The protocols were approved by CAUAP-UFRJ. The technicians dedicated to the animal facility at the Institute of Medical Biochemistry Leopoldo de Meis (UFRJ) conducted all the procedures related to rabbit husbandry under strict guidelines to ensure the careful and consistent handling of the animals. The experiments were conducted using fifth-instar nymphs or adult females of *R*. *prolixus* obtained from a colony at the Institute of Medical Biochemistry Leopoldo de Meis at UFRJ, Brazil. These insects were kept at 28°C and 70% of relative humidity and were fed with rabbit blood at regular intervals of 3 weeks.

### 2.2. Artificial feeding

Blood was collected in syringes containing the following anticoagulants at the indicated final concentrations: citrate [0.42% (w/v), EDTA (10 mM), or heparin (5 U/mL)] from the rabbit’s marginal vein of the ear. The content was mixed gently and used to feed the experimental animals using an artificial apparatus [23].

### 2.3. Analysis of physiological parameters

Fifth instar nymphs were fed as described in section 2.2. The weight of each insect was measured gravimetrically before, immediately, and on days 1, 3, 7, 10, 14, 17, 21, and 24 after feeding. The number of dead and molting insects was also quantified on the same days.

### 2.4. Analysis of reproductive parameters

Females were fed as described in section 2.2. The number of eggs and nymphs was quantified 40 days after feeding.

### 2.5. Parasites and experimental infections

*T*. *cruzi* epimastigotes (Y strain) were cultivated at 28 °C in LIT (liver infusion tryptose) medium [31] supplemented with 10% FCS (Vitrocell, São Paulo, Brazil). Population growth was measured by direct cell counting in a hemocytometer. In all experiments, cells were used in the exponential phase of growth.

Fifth-stage nymphs were artificially fed on heat-inactivated rabbit blood containing 5 × 10^7^ epimastigotes/mL. The protocol for quantification parasites in the insect gut were adapted from [32]. Each infected triatomine was dissected 3, 10, 17, or 24 days post-infection to extract the following three distinct regions of the intestine: the anterior midgut, the posterior midgut, and the hindgut. Samples were homogenized in phosphate-buffered saline (PBS; pH 7.4) and examined by direct microscopic observation. The population density of each *T*. *cruzi* stage in the different regions of the insect gut was quantified using a Neubauer chamber and classified according to their morphological and motility characteristics.

### 2.6. Anticoagulant activity of heparin

Blood was collected from the rabbit’s marginal vein of the ear using syringes containing increasing concentrations of heparin (Hepamax-S, Blausiegel) until the final concentrations of 1, 2, 2.5, 5, 7.5, and 10 U/mL of blood. Then, 1 mL of blood was transferred to a new tube and incubated at 25°C under slow agitation for 30 min. This was followed by centrifugation of the samples at 3.800 × *g* for 10 min at room temperature.

### 2.7. Inhibition of DNA amplification by heparin

Adult females were artificially fed on blood collected with heparin at the final concentrations of 2.5–10 U/mL of blood. After 24 h of feeding, the midgut of the insects was dissected and total DNA was extracted following the protocol described by [33]. The DNA sample was subjected to electrophoresis on 0.8% agarose gel. Purified DNA (20 ng) was used as a template to amplify a 115-bp fragment of the 18S rRNA using the following 18SRt primers: forward 5ʹ-TGTCGGTGTAACTGGCATGT-3ʹ and reverse 5ʹ-TCGGCCAACAAAAGTACACA-3ʹ. Polymerase chain reaction (PCR) was performed using a PCR thermal cycler (model 9700, Applied Biosystems) with an initial denaturation step at 94°C for 5 min, followed by 40 cycles of amplification at 94°C for 30 s, 60°C for 30 s, and 72°C for 30 s, and a final extension step at 72°C for 10 min.

## 3. Results

**Fig 1:**
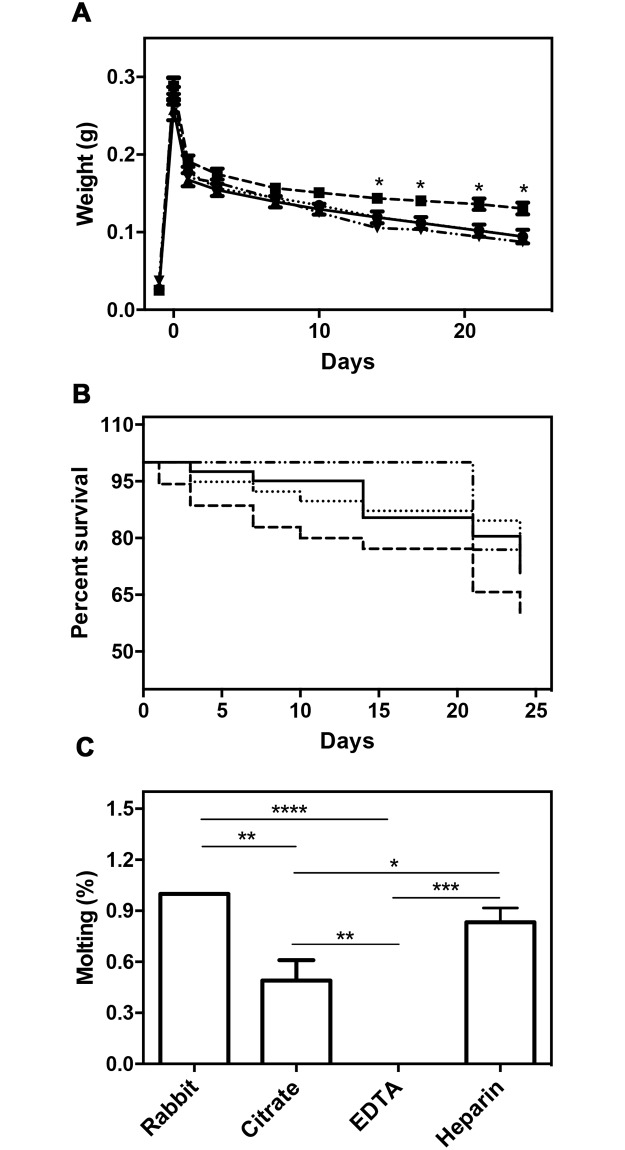
Effects of anticoagulants on blood digestion, survival and molting. The fifth instar nymphs were fed on live rabbit (dotdash line) or on blood collected with citrate (dotted line), EDTA (dashed line), or heparin (continuous line) as anticoagulants. The weight of each insect was measured before and in the following days after feeding them (A), and the number of dead insects was quantified for 24 days (B). On the 24th day after feeding, the percentage of molted insects was calculated (C). Statistical analysis: two-way ANOVA, followed by Bonferroni test (A), total number of insects observed N_Rabbit_ 26, N_citrate_ 39, N_EDTA_ 35, N_Heparin_ 40. Logrank test (B), total number of insects observed N_Rabbit_ 26, N_citrate_ 39, N_EDTA_ 35, N_Heparin_ 40. And one-way ANOVA, followed by Tukey test (C), total number of insects observed N_Rabbit_ 20, N_citrate_ 28, N_EDTA_ 21, N_Heparin_ 27. * P < 0.05; ** P < 0.01; *** P < 0.001; **** P < 0.0001.

*R*. *prolixus* is a hemimetabolous insect that passes through five nymphal stadia until adulthood, when it undergoes maturation of the reproductive system and the complete formation of the wings. During the nymphal stage, the insects can eat up to nine times their own weight during a single blood meal event [34], and each blood meal marks the occurrence of a molting cycle [29]. To determine the influence of anticoagulants on this process, we first analyzed *R*. *prolixus* blood ingestion. We confirmed that the insects in the fifth instar can eat nine to ten times their own weight, which was not influenced by any of the coagulants used. The weight curve, in its turn, was less accentuated in the insects that were fed on blood containing EDTA (Fig 1A). We also analyzed the survival rate and the number of animals that molted during 24 days after feeding. The survival rate of the insects that were fed on EDTA-containing blood was slightly lower after 24 days but was not significantly different compared to the other three groups (Fig 1B). However, when the percentage of molting insects was analyzed, we observed that 80% of the insects that were fed on heparin-containing blood were able to molt compared to 50% of the insects that were fed on blood collected with citrate, whereas none of the insects that were fed on EDTA-containing blood were able to molt (Fig 1C).

**Fig 2:**
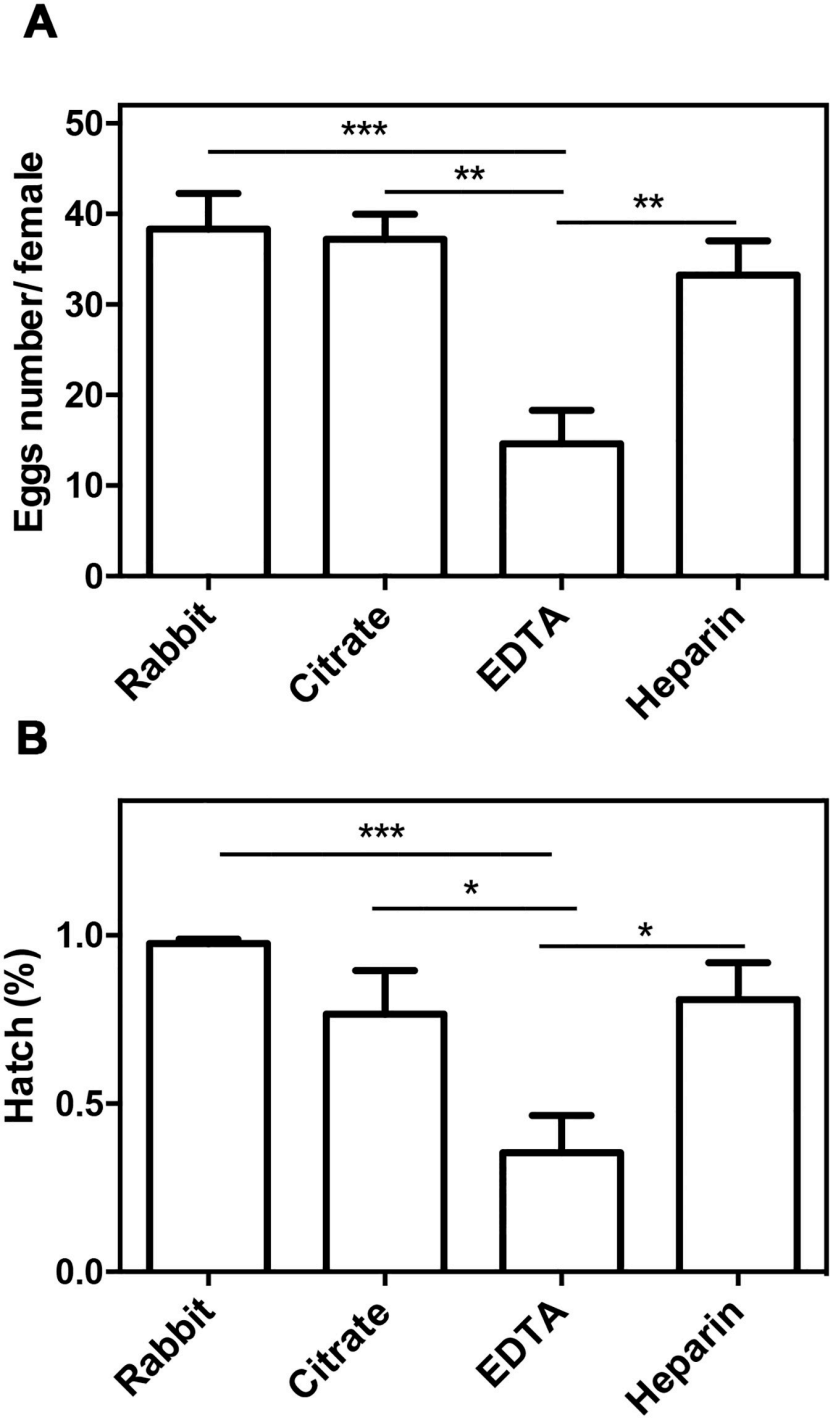
Effects of anticoagulants on fertility. Females were fed on live rabbit or artificially on blood collected with citrate, EDTA, or heparin as anticoagulants. After 40 days, the number of eggs (A) and nymphs was quantified, and the hatch percentage was calculated (B). Statistical analysis: one-way ANOVA, followed by Tukey test, total females number observed N_Rabbit_ 12, N_citrate_ 10, N_EDTA_ 8, N_Heparin_ 12 (A), and total number of eggs observed N_Rabbit_ 470, N_citrate_ 372, N_EDTA_ 117, N_Heparin_ 399. * P < 0.05; ** P < 0.01; *** P < 0.001.

Blood meal is also essential for the reproduction of *R*. *prolixus*. During adulthood, each blood meal synchronizes the reproductive cycle, and the quality and quantity of blood that the insect ingests interfere directly in the development and production of eggs [18,34]. Around the third day after the blood meal, the ovaries of *R*. *prolixus* females are filled with oocytes, and then oviposition begins on the fifth day [35]. First-instar nymphs hatch in a period of 10–20 days after egg laying. *R*. *prolixus* females were fed on blood collected with citrate, EDTA, or heparin, placed in individual flasks and were kept for 40 days. On the 40th day after feeding, the laid eggs and nymphs were counted in each flask. The total number of eggs was similar in the insects that were fed on live animals or on blood collected with heparin and citrate, but this number was reduced to half in females that were fed on EDTA-containing blood (Fig 2A). Furthermore, the percentage of hatched eggs was lower in this group (Fig 2B). The eggs laid by the females that were fed on EDTA-containing blood showed abnormalities such as dryness or dehydration, hatching interruption, and malformation of the nymph (S1 Fig).

**Fig 3:**
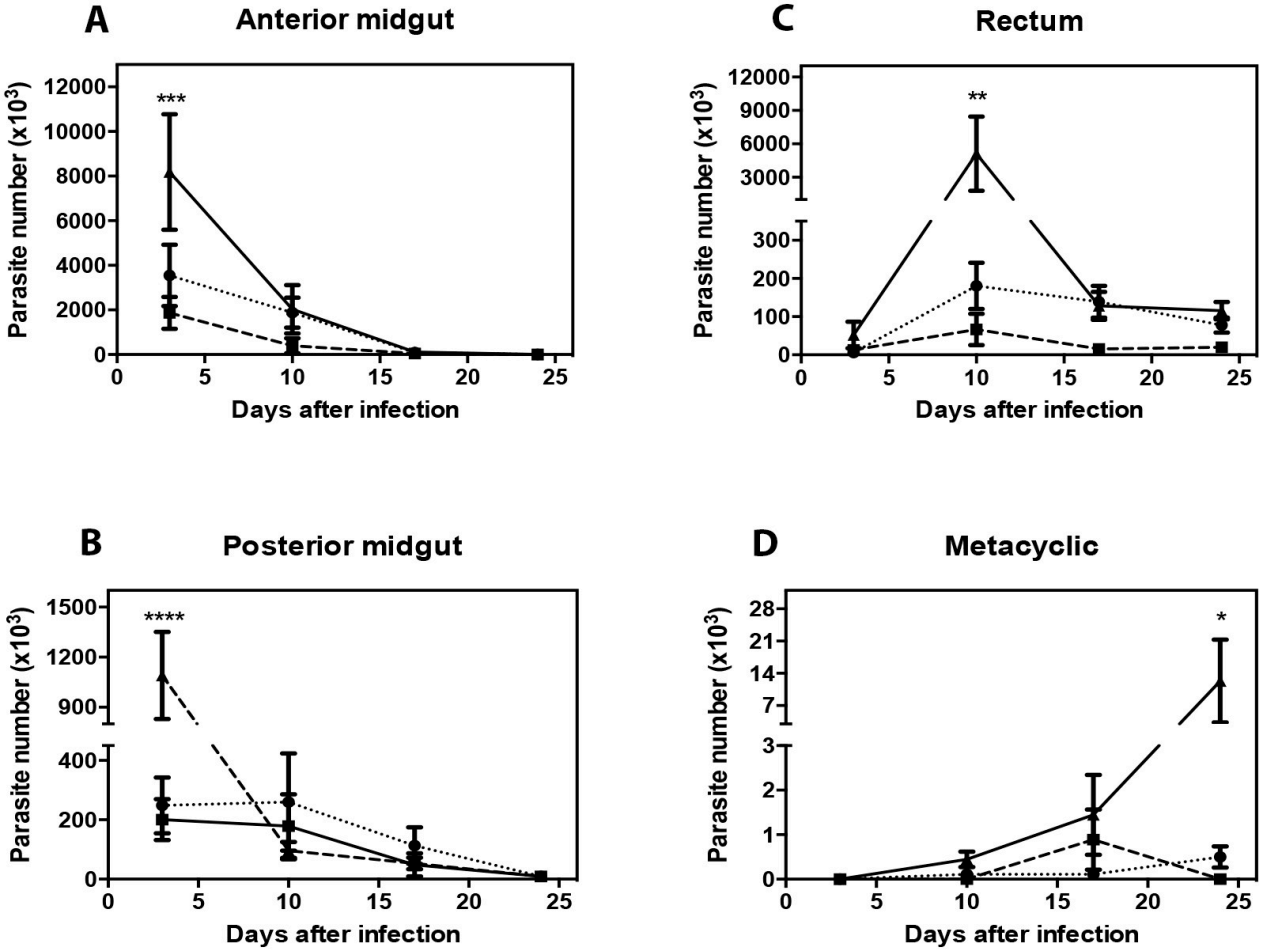
Effects of anticoagulants on *Trypanosoma cruzi* infection. Fifth instar nymphs were fed on blood collected with citrate (dotted line), EDTA (dashed line), or heparin (continuous line) as anticoagulants containing 10^7^ parasites/mL. The infection profile was obtained by counting the parasite numbers in the anterior midgut (A), posterior midgut (B), and rectum (C). The number of metacyclic trypomastigotes was measured in the rectum (D). Statistical analysis: two-way ANOVA, followed by Bonferroni test, Total number of insects observed N_citrate_ 9, N_EDTA_ 9, N_Heparin_ 9. * P < 0.05; ** P < 0.01; *** P < 0.001; **** P < 0.0001.

Several research groups have analyzed the interaction between triatomine and trypanosomatids, wherein artificial vector infection has been considered important because it eliminates the need to keep warm-blooded animals infected with the parasite. We analyzed the effect of different anticoagulants (citrate, EDTA, and heparin) on the development of *T*. *cruzi* infection in the intestine of *R*. *prolixus*. The number of parasites present in the anterior midgut (Fig 3A) and the posterior midgut (Fig 3B) was higher in the insects that were fed on heparin-containing blood compared to the other two groups. There was also a prominent increase, compared to the two other groups, in the number of parasites on the 10th day after infection in the rectum of insects that were fed on blood collected with heparin (Fig 3C). Metacyclogenesis, a process in which epimastigotes found in the digestive tract transform into metacyclic trypomastigotes that could infect the vertebrate host, occurs in the rectum. Therefore, in addition to the total number of parasites present in the rectum, we analyzed the number of metacyclic trypomastigotes present in the rectum. We observed that the number of metacyclic trypomastigotes was about 15 times higher in the animals that were fed on blood containing heparin (Fig 3D). To test if the anticoagulants used affect the parasites directly we analyzed the *T*. *cruzi* (Y) growth curve and test their viability in the presence of each anticoagulant used (Citrate, EDTA and Heparin) (S2 Fig). We observed no changes when compared the treatments with a control in the absence of any anticoagulant. So, these differences in infection curves apparently are not related to a direct effect of the anticoagulants used against the parasites.

**Fig 4:**
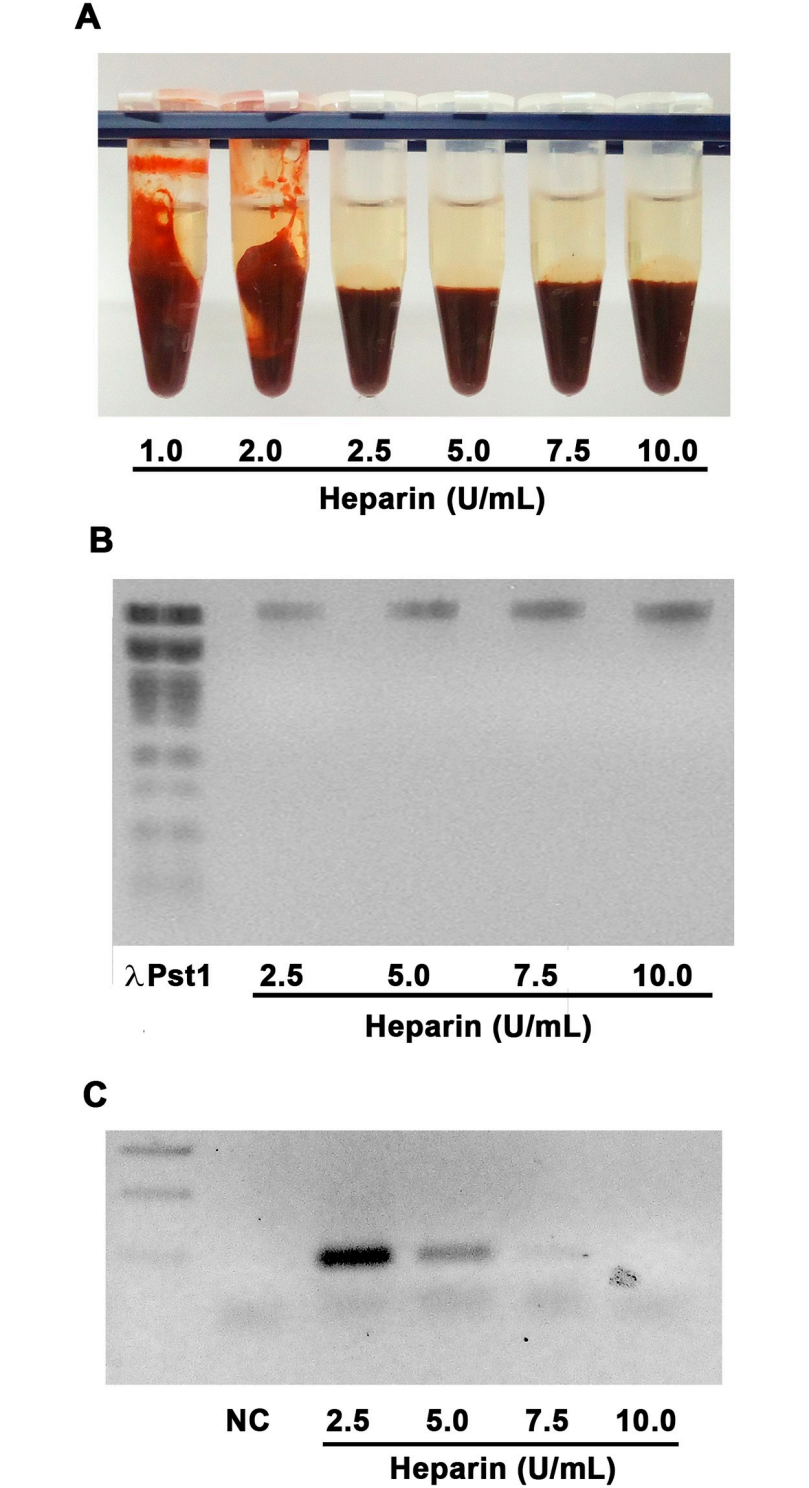
Heparin tests. Rabbit blood was collected with heparin at different concentrations, incubated for 30 min, and centrifuged (A). DNA was purified from the guts of adult females that were fed on blood collected with heparin at different concentrations (B). Purified DNA was used as the template in a polymerase chain reaction using 18S RNA primers (C). NC, negative control.

Citrate, EDTA, and heparin are the most common anticoagulants used to stabilize the blood collected for feeding hematophagous insects. However, as molecular biology experiments have become more popular, the use of heparin has been reconsidered as it causes an inhibitory effect on Taq DNA polymerase. To validate the use of heparin in molecular biology research on hematophagous insects, we first defined the units required to inhibit blood coagulation. Heparin unit is defined as the amount required to inhibit coagulation of 1 mL of sheep blood. To inhibit rabbit blood coagulation under experimental conditions, we used 2.5 units of heparin (Fig 4A). The blood collected with increasing concentrations of heparin was used to feed the hematophagous insect *R*. *prolixus*. After 24 h, the gut tissue and its contents were collected, and DNA purification was performed, by which the entire genomic DNA was obtained (Fig 4B). Then, DNA was used as a template in PCR. We were able to amplify the DNA extracted from the insects that were fed on blood containing 5 units of heparin/mL; whereas increasing heparin concentrations drastically inhibited DNA amplification (Fig 4C).

## 4. Discussion

Hematophagous insects are capable of transmitting disease agents to humans during the feeding process. Therefore, understanding the physiology of blood meal feeding is essential for the development of control strategies. Several studies have used artificial feeding processes to provide the insect with a defined diet [36] or to reduce the use of living hosts [37,38]. In this study, we compared the survival rate, molting efficiency, fertility, and infection development between insects that were fed on blood collected with three different anticoagulants (citrate, EDTA, and heparin). We observed an equal increase in the weight of *R*. *prolixus* insects that were fed on blood containing all the tested anticoagulants (Fig 1A). This result is in agreement with that reported by [39], who demonstrated an increase of 10 times in the weight of fifth instar insects that were fed on a blood meal when compared to unfed insects. No difference on the amount of blood ingested was also observed in *Triatoma infestans* third instar nymphs fed artificially on citrated blood or heparinized [40]. However, some anticoagulants may affect feeding efficacy in particular hematophagous species. *Rhipicephalus appendiculatus* nymphs, for example, did not feed on acid citrate dextrose or EDTA treated blood but did feed to repletion on defibrinated or heparinized blood [41]. *Amblyoma variegatum* ticks interrupted feed and detached after 48h when fed on blood treated with EDTA, while ticks fed artificially on heparinized or defibrinated blood totally engorged [42]. Despite the full engorgement, *R*. *prolixus* nymphs fed on blood containing EDTA showed a less pronounced decline in their weight over time when compared to the other two groups (Fig 1A), which is probably related to the lower capacity of blood digestion. Citrate, EDTA, and heparin are frequently used as anticoagulants, and each one has different effects on the coagulation cascade. Heparin binds to antithrombin, a serine protease inhibitor, altering its conformation and increasing the inhibition of thrombin (Factor IIa) and other serine proteases from blood coagulation cascade. As thrombin stimulates the conversion of fibrinogen to fibrin its inhibition, consequently, diminish the fibrin formation. EDTA and citrate affect the coagulation cascade by chelating calcium ions that are necessary for factor IX activation in the intrinsic pathway and factor VII via the extrinsic pathway. By acting as chelators, the anticoagulants EDTA and citrate may alter the course of the insect’s digestion process, due to the presence of digestive enzymes that depend on ions for their activity, such as aminopeptidases and carboxypeptidases [43,44].

An impaired digestion could lead to a change in nutrient uptake by the insect, followed by alterations in molting, metamorphosis, and reproduction as these processes depend on the quality and quantity of the ingested nutrients [12,45–47] as shown by the molting inefficiency in our study (Fig 1C). Similar findings were reported for artificially fed *A*. *variegatum* whose molting success of heparinized and defibrinated blood fed ticks was comparable to those fed on cattle [42]. Previous studies show that *T*. *infestans* first instar nymphs fed on defibrinated, heparinized or citrated blood has a similar molt percentage to second instar nymphs (75–85%). Although, when the insects were maintained on that artificially feed, the molt percentage diminished. Third instar nymphs fed on heparinized blood are 20% more efficient on molting than nymphs fed on citrated blood, and 40% than nymphs fed on defibrinated blood [48]. Despite the molting inefficiency, we could not observe differences in the survival curves between the insect groups (Fig 1B). Nevertheless, as insects that were fed on blood containing EDTA and citrate were not able to digest efficiently all the blood ingested during the blood meal, we could expect that they will not survive for the same time as that of the insects that were able to perform the molting process. To observe this phenomenon in a better manner, it is necessary to follow up the insects for a longer period of time than that in the present study. A decreased of survival was observed in *Glossina austeni* fed on blood collected with sodium citrate (68%) when compared with flies fed on defibrinated blood (95%). However, the same is not observed for *Glossina brevipalpis*, that survival was not significantly different between these groups [49].

Total number of eggs and hatch percentage of eggs laid by insects that were fed on live rabbit, citrated- or heparinized-treated blood was not significantly different in our experiments, and hatch percentage is similar to the reported before, reaching 95% [39]. Previous studies on artificial feeding using *A*. *variegatum* as a model showed that egg-laying of heparinized and defibrinated blood fed ticks was comparable to those fed on cattle [42], as we observed in *R*. *prolixus* fed on heparinized blood. Even as, no differences in feeding efficiency or fertility were found in a direct comparison of *Cimex lectularius* maintained under artificial (1% heparinized blood) or natural feeding, but analysis of the full lifecycle revealed that artificially fed bedbugs became significantly smaller and laid fewer eggs than rodent-fed bed bugs [50]. Our results showed no significative difference between eggs number and hatch of insects fed on citrated and heparinized blood, however, previous studies on artificial feeding using *T*. *infestans* demonstrated that bugs laid more viable eggs when fed on heparinized blood in comparison to bugs fed on sodium oxalate-, sodium citrate-, or sodium-fluoride-treated blood. Defibrinated and heparinized blood were effective anticoagulants for use in preparing blood for Glossina *sp*. However, sodium citrate and EDTA were not suitable when analyzing parameters related to longevity and reproduction (as abortion, pupae weight/size and number, and insemination) [49,51,52]. Adult insects that were fed on EDTA-containing blood laid a lower number of eggs (Fig 2A), resulting in a lower egg hatch percentage (Fig 2B); in other words, the embryonic development appears to be affected by this anticoagulant. Studies on *Drosophila melanogaster* have shown the importance of calcium signaling in development. Homozygous mutations in the genes affecting calcium channels and other proteins involved in the signaling of this ion are often lethal in the early stages of development [53], which could be related to the reduced fecundity and egg hatching capacity of insects fed on blood containing EDTA and citrate.

The digestive system of *R*. *prolixus* is composed of three distinct portions, the foregut; the midgut that is divided in two portions, anterior midgut (stomach, AM) and the posterior midgut (PM); and the hindgut (rectum). The ingested blood is stored, concentrated, and hemolyzed in the AM of bugs, and factors such as insect’s produced molecules and bacterial interaction could determinate the success of the *T*. *cruzi* infection when they are obtained during a blood meal. Therefore, 24 h after the ingestion of trypanosomatids, the first intermediate form can be found in the PM. In this intestinal portion, where blood is digested, epimastigotes attach to perimicrovillar membranes and multiply. *T*. *cruzi* adhesion onto the rectal wall and the nutritional stress in the rectal ampulla play a role in metacyclogenesis [54]. When we delivered *T*. *cruzi* parasites in blood collected with citrate and EDTA to the insects, at later time periods after blood ingestion, we observed a lower number of parasites in the AM, PM and rectal ampulla compared to the heparin group (Fig 3A–3C). In addition, these insects showed a lower number of metacyclic trypomastigotes in the rectum (Fig 4D). These data demonstrate that some anticoagulants may affect the parasite development inside the arthropod host, and, therefore, their use in parasite–vector interaction studies would not be appropriate. Previous studies have shown that the nutritional status of the vector has an extreme impact on *T*. *cruzi* development [55,56]. When insects fed on blood containing EDTA exhibited an impaired digestion, the alterations in the microenvironment of the gut might have influenced the parasite numbers as well. Moreover, a study demonstrated that EDTA was able to interfere in the development of the sporogonic cycle of *Plasmodium vivax* in *Anopheles* mosquitoes fed on blood containing EDTA. The mosquitoes showed lower mean oocyst numbers in comparison to those fed on blood containing heparin. According to the authors of that study, this result could be explained by the fact that microgametocytes require Ca^2+^, Mn^2+^, and Mg^2+^ to activate important enzymes for the process of exflagellation and for the motility of ookinetes [57].

PCR is a current basic procedure in experimental research. Sodium heparin, an anticoagulant widely used to stabilize the collected blood, has been known to inhibit DNA polymerase activity in PCR assays. The degree of heparin inhibition depends on its concentration contained in the nucleic acid preparation [58–61]. One of our concerns regarding heparin use as an anticoagulant is DNA polymerase inhibition. Therefore, we tested different heparin concentrations to define the best concentration. Concentrations between 2.5 and 5 U/mL were able to inhibit coagulation without severely impairing DNA polymerase activity and should be considered as the range of use for feeding experiments. Future screenings should analyse the concentration between 2.5 and 5 U/mL to observe the maximum concentration that could be use without any loss in DNA polymerase activity. In that moment, based on our set of experiments the concentration of 2.5 U/mL of heparin is the more indicate.

Previous works demonstrated that blood containing high levels of heparin (10%) was unsuitable for artificial rearing of bed bug colonies, whereas bed bugs fed on 1% heparinized blood and those that naturally ingested rat blood completed their life cycle with no significant differences in mortality [50]. However, the bugs became significantly smaller and laid fewer eggs than those laid by rodent-fed bed bugs. A previous report by [62] on *R*. *prolixus* showed that citrated blood is better than heparinized blood for colony maintenance and that oxalates and fluorides can be deleterious for the insects. However, the units or the percentage of heparin used in the that report was not clear. The results of our study on *R*. *prolixus* demonstrated that among the three anticoagulants tested, EDTA had the highest negative effect on all conditions evaluated. And based on our findings we suggest the use of heparin as the anticoagulant of choice for *R*. *prolixus* feeding experiments.

## Supporting information

S1 Fig Eggs from females fed on blood containing different anticoagulants are morphological differents. Females were fed on live rabbit or artificially on blood collected with citrate, EDTA, or heparin as anticoagulants. After 40 days, the eggs were collected. The eggs laid by the females that were fed on EDTA-containing blood showed abnormalities such as dryness or dehydration, hatching interruption, and malformation of the nymph. Total number of eggs observed N_Rabbit_ 470, N_citrate_ 372, N_EDTA_ 117, N_Heparin_ 399.
(TIF)

S2 Fig Viability analysis of *T*. *cruzi* epimastigotes previously treated during three days with citrate, heparin and EDTA was assessed by incorporation of PI and flow cytometry. Control parasites (A), Dead parasites (B), parasites treated with 0.42% (w/v) citrate (C), parasites treated with 10 mM EDTA (D), parasites treated with 5 U/mL heparin (E), and percentage of live and dead epimastigotes in different treatment conditions (F). Parasite survival after treatment with anticoagulants at same concentrations as in viability assay was monitored by growth curve (G).
(TIF)
