# Supplementary figures and images for: Evaluating the effects of anticoagulants on Rhodnius prolixus artificial blood feeding
Source: PLoS One. 2018 Nov 29;13(11):e0206979. doi: 10.1371/journal.pone.0206979 (PMC6264878; doi:10.1371/journal.pone.0206979)

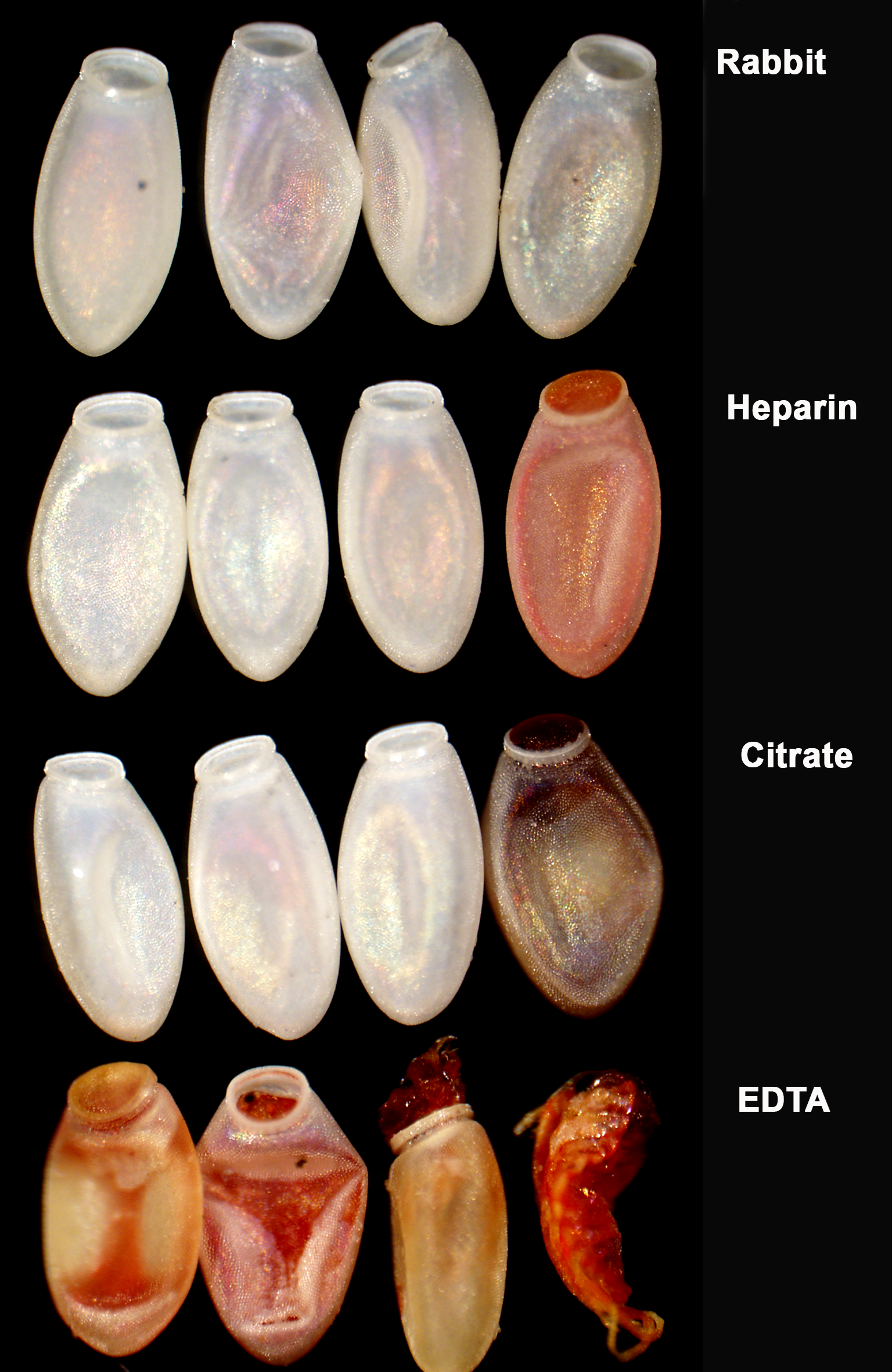

Supplement: S1 Fig — Females were fed on live rabbit or artificially on blood collected with citrate, EDTA, or heparin as anticoagulants. After 40 days, the eggs were collected. The eggs laid by the females that were fed on EDTA-containing blood showed abnormalities such as dryness or dehydration, hatching interruption, and malformation of the nymph. Total number of eggs observed NRabbit 470, Ncitrate 372, NEDTA 117, NHeparin 399. (TIF) [file pone.0206979.s001.tif]

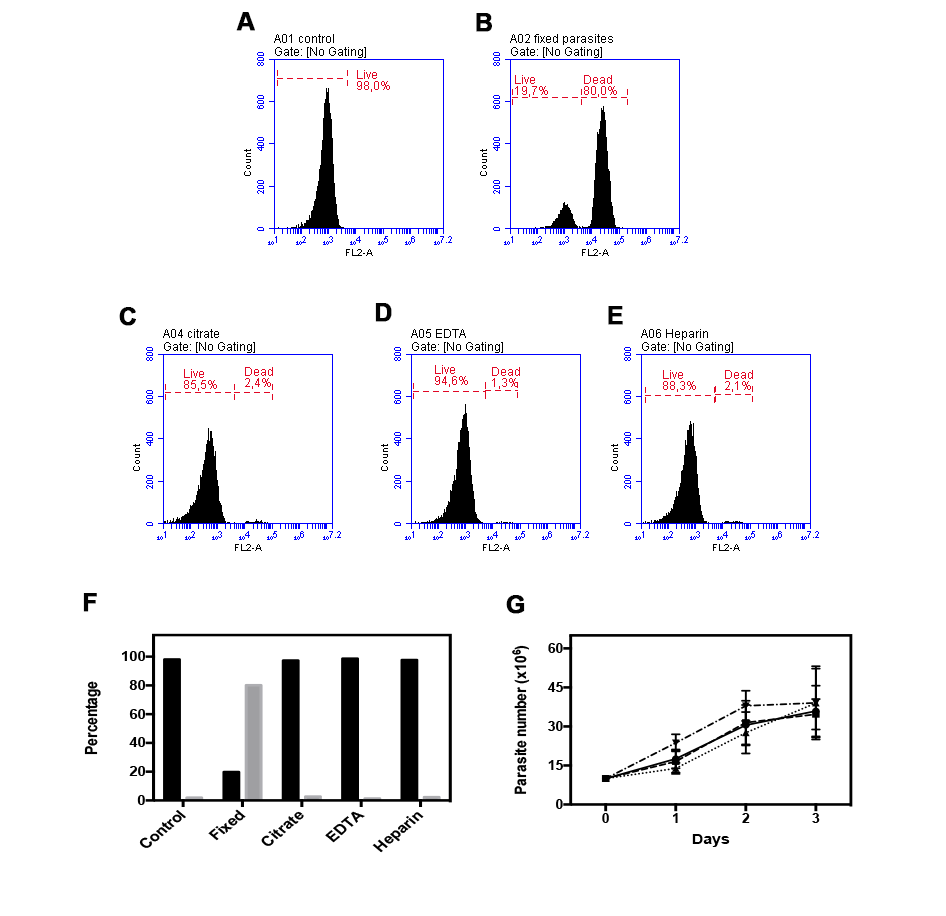

Supplement: S2 Fig — Control parasites (A), Dead parasites (B), parasites treated with 0.42% (w/v) citrate (C), parasites treated with 10 mM EDTA (D), parasites treated with 5 U/mL heparin (E), and percentage of live and dead epimastigotes in different treatment conditions (F). Parasite survival after treatment with anticoagulants at same concentrations as in viability assay was monitored by growth curve (G). (TIF) [file pone.0206979.s002.tif]
